# Exome Capture Sequencing of Adenoma Reveals Genetic Alterations in Multiple Cellular Pathways at the Early Stage of Colorectal Tumorigenesis

**DOI:** 10.1371/journal.pone.0053310

**Published:** 2013-01-02

**Authors:** Donger Zhou, Liu Yang, Liangtao Zheng, Weiting Ge, Dan Li, Yong Zhang, Xueda Hu, Zhibo Gao, Jinghong Xu, Yanqin Huang, Hanguang Hu, Hang Zhang, Hao Zhang, Mingming Liu, Huanming Yang, Lei Zheng, Shu Zheng

**Affiliations:** 1 The Key Laboratory of Cancer Prevention and Intervention of China National Ministry of Education, the Key Laboratory of Molecular Biology in Medical Sciences of Zhejiang Province, Cancer Institute, Hangzhou, Zhejiang, China; 2 The Second Affiliated Hospital of Zhejiang University School of Medicine, Hangzhou, China; 3 Beijing Genomics Institute (BGI)-Shenzhen, Shenzhen, China; 4 Department of Oncology and Department of Surgery, The Sidney Kimmel Comprehensive Cancer Center at Johns Hopkins, Johns Hopkins University School of Medicine, Baltimore, Maryland, United States of America; Howard University, United States of America

## Abstract

Most of colorectal adenocarcinomas are believed to arise from adenomas, which are premalignant lesions. Sequencing the whole exome of the adenoma will help identifying molecular biomarkers that can predict the occurrence of adenocarcinoma more precisely and help understanding the molecular pathways underlying the initial stage of colorectal tumorigenesis. We performed the exome capture sequencing of the normal mucosa, adenoma and adenocarcinoma tissues from the same patient and sequenced the identified mutations in additional 73 adenomas and 288 adenocarcinomas. Somatic single nucleotide variations (SNVs) were identified in both the adenoma and adenocarcinoma by comparing with the normal control from the same patient. We identified 12 nonsynonymous somatic SNVs in the adenoma and 42 nonsynonymous somatic SNVs in the adenocarcinoma. Most of these mutations including OR6X1, SLC15A3, KRTHB4, RBFOX1, LAMA3, CDH20, BIRC6, NMBR, GLCCI1, EFR3A, and FTHL17 were newly reported in colorectal adenomas. Functional annotation of these mutated genes showed that multiple cellular pathways including Wnt, cell adhesion and ubiquitin mediated proteolysis pathways were altered genetically in the adenoma and that the genetic alterations in the same pathways persist in the adenocarcinoma. CDH20 and LAMA3 were mutated in the adenoma while NRXN3 and COL4A6 were mutated in the adenocarcinoma from the same patient, suggesting for the first time that genetic alterations in the cell adhesion pathway occur as early as in the adenoma. Thus, the comparison of genomic mutations between adenoma and adenocarcinoma provides us a new insight into the molecular events governing the early step of colorectal tumorigenesis.

## Introduction

Colorectal cancer (CRC) is the third most commonly diagnosed cancer in males and the second in females in the world [1]. In China, the incidence of CRC has been rising in the most recent years [2]. New insights into the pathogenesis of this lethal disease are needed. The tumorigenesis of CRC is characterized by a multi-stage pathological evolution process. Most of colorectal adenocarcinomas are believed to arise from adenomas, which are premalignant lesions. The CRC development involves multiple genetic alterations including both oncogenic mutations and loss of tumor suppressor genes [3], [4]. Among these genetic alterations, inactivation of the APC gene was often detected in small adenomas, the early stage of CRC development. KRAS mutations were detected when a small adenoma grows into a large adenoma (>1 cm diameter) [5]; and alterations in PIK3CA and TP53 or other genes [6]–[9] occurred during the development of invasive adenocarcinoma. With advances in sequencing technology, the whole exomes and even the whole genomes of individual colorectal adenocarcinomas have been sequenced and a comprehensive landscape of genetic alterations was delineated [10]–[12].

Patients with colorectal adenomas are at increased risk of developing colorectal cancer. The risk of metachronous neoplasm including adenocarcinoma can be predicted by the size and the pathology of initial adenomas after their initial polypectomy [13]. Adenomas have been frequently identified since colonoscopy was used for routine colorectal cancer screening. Nonetheless, the pathologic features of initial adenoma cannot adequately predict the occurrence of adenocarcinoma. Sequencing the whole exome of the adenoma will help understand the genetic characteristics of the adenoma and potentially identify molecular biomarkers that can predict the occurrence of adenocarcinoma more precisely. It will also help understand the molecular mechanisms underlying the initial stage of colorectal tumorigenesis and may potentially uncover the pathways that may be targeted to stop the tumorigenesis process at its early stage.

In this study, we performed the exome capture sequencing of normal mucosa, adenoma and adenocarcinoma tissues from the same CRC patient. Somatic single nucleotide variations (SNVs) were identified in both the adenoma and the adenocarcinoma by comparing with the normal control from the same patient. Similar enrichment in nucleotide transitions was found in the adenoma and the adenocarcinoma while the mutation rate in the adenoma was lower than that in the adenocarcinoma. Functional annotation of the mutated genes showed several pathways including Wnt pathway, cell adhesion pathway and ubiquitin mediated proteolysis pathway were altered genetically in the early stage of colorectal tumorigenesis. The comparison between adenoma and carcinoma provides us a new insight into the molecular events governing the early step of CRC tumorigenesis.

## Materials and Methods

This study was proved by the Ethical Committee of the 2nd Affiliated Hospital Zhejiang University School of Medicine and Zhejiang University Cancer Institute. For human tissue samples, written informed consent was obtained in compliance with the consent procedure approved by the Ethical Committee.

### Patient Samples

Initial tumor specimens were obtained from a 66-year-old Chinese man with no family history of CRC who underwent a resection of colon cancer. Two polyps locating about 4 cm near the primary tumor in the resected colon were also resected. Part of the primary tumor and one of the polyps were dissected and snap frozen in liquid nitrogen. The resected adenocarcinoma was well to moderately differentiated and invaded into sub-mucosa without regional lymph node metastasis. The resected polyp was a tubular adenoma. Portions of the patient’s adenocarcinoma, adenoma, normal mucosa and peripheral blood were banked for exome capture sequencing and digital gene expression profiling. Additional 215 cases of fresh frozen tissues of adenocarcinoma were obtained from the tissue bank of Zhejiang University Cancer Institute. Seventy-three pairs of formaldehyde fixed-paraffin embedded (FFPE) tissues of matched adenoma and adenocarcinoma from the same patients were obtained from the pathology department of the 2^nd^ affiliated hospital of medical school of Zhejiang University. These additional specimens were used as a validation sample set. All the tissue samples were reviewed under microscope by 2 pathologists and the tumor contents of the adenoma and adenocarcinoma samples were more than 70%.

### DNA and RNA Extraction

DNA was extracted from fresh frozen tissues using a DNeasy Blood & Tissue Kit (Qiagen, Germany) and from FFPE tissues using a QIAamp DNA FFPE Tissue Kit (Qiagen, Germany) according to the manufacturer’s instructions. RNA was extracted from fresh frozen tissues using TRIzol (Invitrogen, CA, USA) according to the manufacturer’s instructions. DNA without whole genomic amplification was directly used for exome capture.

### Public Genome Data

The human reference genome sequences (NCBI build 36.1/hg18) were downloaded from the UCSC (University of California, Santa Cruz) database (http://genome.ucsc.edu). Human reference mRNA sequences (Refseqs of coding mRNA) were downloaded from NCBI (http://www.ncbi.nlm.nih.gov/RefSeq). Known SNPs (single nucleotide polymorphism) were downloaded from the UCSC database (single nucleotide polymorphism database, dbSNP version 132).

### Exome Capture, Sequencing and Mapping

Exome capture was performed using the NimbleGen 2.1 M Human Exome Array (Roche NimbleGen, WI, USA) which includes 180,000 coding exon (28.4 Mb) and adjacent regions (5.7 M) of one human genome (http://www.nimblegen.com/products/seqcap/arrays/exome/). Genomic DNA was randomly fragmented by nebulization to an average size of 500 bp. A pair of linkers was ligated to each end of DNA fragments. The linker-ligated DNA fragments were then hybridized to the NimbleGen 2.1 M Human Exome Array. Unbound fragments were washed away; and the target-enriched DNA was kept and then eluted. Enriched samples were amplified by ligation-mediated PCR (LM-PCR).

The captured DNA fragments were randomly ligated by DNA ligase. The ligated long exon-enriched DNA was sheared to small pieces in about 200 bp on average, and then ligated with Illumina compatible adapters. The resulted DNA fragments were subjected to the standard Solexa library preparation. The exome-enriched shotgun library was sequenced at the Illumina GAII platform (Illumina, CA, USA). Raw data from GA sequencing were processed by the Illumina Pipeline v1.3.1 software. The low quality reads were discarded (fractions of N bases > = 0.1 and fractions of bases with quality less than 5> = 0.5). The clean reads were aligned against the human reference genome (NCBI build 36.1/hg18) using the Burrows-Wheeler Alignment tool (BWA) [14]. The alignment results were further processed sequentially with local realignment, duplicate read marking, and base quality recalibration by using the Picard (developed at the Broad Institute [15]) and GATK [16], [17] pipeline software.

The chi-square test was used for comparison of the enrichment in nucleotide transitions and mutation rate between the adenoma and the adenocarcinoma.

### Data Deposition and Availability

Raw data (Fastaq sequence) has been uploaded to the Short Read Archive database and the accession number is SRA052805.

### Somatic Single Nucleotide Variation Analysis

Somatic single nucleotide variations of the adenoma and the adenocarcinoma were called using SAMTools (The Sequence Alignment/Map Tools) [18]. The following filtering criteria were applied: 1) the CLR score of each mutation must be more than 15; 2) only 1 alternative allele supporting read is allowed in normal control; 3) there should be at least 4 alternative allele supporting reads in tumor (adenoma or adenocarcinoma); 4) the total number of MQ0 reads in normal and tumor should be no more than 1. Finally, the mapped reads of all the SNVs were manually reviewed by using the Integrative Genomics Viewer (IGV) [19].

### Digital Gene Expression Profiling

Digital gene expression profiling was applied using deep sequencing of cDNA tags [20], [21]. The libraries for sequencing tags were prepared using Illumina Gene Expression Sample Prep Kit (Illumina, CA, USA) according to manufacturer’s instruction. Briefly, Messenger RNA was purified using Oligo(dT) magnetic beads (Illumina, CA, USA) from total RNA. The first and second-strand cDNA were synthesized using Oligo(dT) as the primer. The bead-bound cDNA was digested with restriction enzyme *Nla*III (Illumina, CA, USA), which recognized and cut the CATG sites. Then, the Illumina adaptor 1 was ligated to the sticky 5' end of the digested fragments. *Mme*I (an endonuclease provided by Illumina with a different recognition site and a digestion site) cut at 17 bp downstream of the CATG site and subsequently produced tags with adaptor 1. After removing 3' fragments, Illumina adaptor 2 was ligated to the 3' ends of tags, thereby acquiring tags with different adaptors at both ends to form a tag library. Finally, the tag libraries were sequenced on an Illumina GA II platform.

Raw sequencing data were processed by removing adapters and low quality reads. All the clean tags were mapped to the reference sequences (NCBI build 36.1/hg18). The number of unambiguous clean tags was calculated for each gene and normalized to TPM (number of transcripts per million clean tags) [20], [21].

### Sanger Sequencing and Sequenom Validation

All the somatic single nucleotide mutations were validated using both Sanger sequencing and Sequenom iPLEX genotyping system (Sequenom Inc., CA, USA).

Primers for amplification and sanger sequencing validation were designed by using an online tool, Primer3 (http://frodo.wi.mit.ed/primer3/), to target regions flanking point mutations and were listed in Table S5. PCR amplifications were performed using HotStarTaq Plus DNA Polymerase (Qiagen, Germany) following manufacturer’s instruction. Sanger sequencing was performed on an ABI 3730 Capillary DNA Analyzer. Sequence trace files were manually analyzed for point mutations.

For Sequenom iPLEX genotyping, all the steps were performed following the manufacturer’s application guide (Sequenom Inc., CA, USA). Briefly, PCR amplification primers and extension probe were designed using the Sequenom MassARRAY Assay Design 3.0 software. PCR amplification and single-base extension were performed following manufacturer’s protocols (Sequenom Inc., CA, USA). After removing residual salt, the purified extension products were dispensed onto a 384-element SpectroCHIP bioarray (Sequenom Inc., CA, USA). SpectroCHIPs were analyzed by a matrix-assisted laser desorption/ionization–time of flight (MALDI-TOF) mass spectrometer. The data was processed and analyzed using MassARRAY Workstation (version 3.3).

## Results

### Sequence Coverage, Analysis of Mutations and Digital Gene Expression Profiling

Exome capture was performed on the normal mucosa, adenoma, and adenocarcinoma tissues from the same patient by using NimbleGen 2.1 M Human Exome Array. The target regions of exome capture include 180,000 coding exon (28.4 Mb) and adjacent regions (5.7 M). Combining parallel sequencing on an Illumina GAII platform, we generated approximately 4.6, 4.4 and 4.3 billion bases of effective sequence data with an average read length of 69 bases for normal mucosa, adenoma and carcinoma, respectively. After mapping to the human reference genome (NCBI36.1/HG18) using Burrows-Wheeler Alignment tool (BWA) [14], we obtained the average depth of each base in the target regions as 46.69×, 46.44× and 45.12× (Table 1) for each sample. The coverage of target regions was more than 98% in all the three samples including adenocarinoma, adenoma, and normal mucosa.

**Table 1:** Summary of sequencing coverage of the normal mucosa, adenoma and adenocarcinoma from the same patient.

Category	Normal Mucosa	Adenoma	Adenocarcinoma
Total raw reads	72586262	68993041	70103375
Mapping rate	91.70%	90.70%	92.70%
Total effective reads	66535567	62548877	65016433
Total effective yield(Mb)	4603.18	4350.15	4456.06
Average read length(bp)	69.18	69.55	68.54
Average sequencing depth on target	46.69	46.44	45.12
Base covered on target	33627496	33597920	33559156
Coverage of target regions	98.60%	98.50%	98.40%

All the mapped sequences (in the target regions and near the target regions) were used for the mutation analysis. Comparing with the sequences data of normal mucosa as the normal control, somatic single nucleotide variations (SNVs) in adenoma and adenocarcinoma were called by SAMtools [18]. We further selected the somatic SNVs in the coding sequence (CDS) of adenoma and adenocarcinoma by excluding those SNVs in flanking sequences like splicing sites, 5′-untranselated regions (UTR), 3′-UTR, introns and intergenic regions. After filtering against the single nucleotide polymorphism database (dbSNP 132) (Table S1), the numbers of the potential nonsynonymous somatic SNVs in the adenoma and adenocarcinoma prior to being validated by the Sanger sequencing and Sequenom genotyping were 16 and 44, respectively.

Simultaneously, digital gene expression profiling of the three samples including adenocarcinoma, adenoma, and normal mucosa were performed with tag based RNA sequencing. The number of clean tags obtained from these three samples was 7492596, 5291578 and 3318930, respectively. All the tags were mapped to the reference genome and only the unambiguous mapped tags were counted for each gene. The differentially expressed genes were selected through adenocaicinoma and adenoma compared with normal mucosa. The differential expressed genes in adenoma were enriched in 9 functional pathways including ribosome, cell adhesion molecules, and peroxisome proliferator-activated receptors (PPAR) signaling pathway (p<0.01) while the differential expressed genes in carcinoma were enriched in 24 pathways (p<0.01) (Table S3 and S4) by using the KEGG pathway analysis tool [22].

### Mutation Spectrum of the Adenoma and the Adenocarcinoma from the same Patient

**Figure 1:**
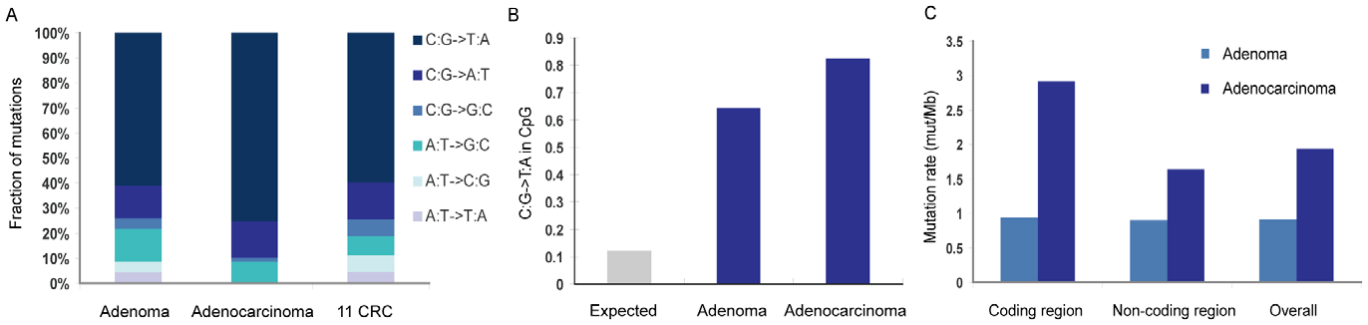
Somatic SNVs pattern in the adenoma and the adenocarcinoma. (A) Somatic mutation spectrum in adenoma and adenocarcinoma, similar with 11 colorectal cancers previously reported [10], [11]. (B) Fractions of guanine mutations at 5′-CpG-3′ dinucleotides in the exome of adenoma and adenocarcinoma. (C) Prevalence of somatic SNVs in the coding region and non-coding region of the exome of the adenoma and the adenocarcinoma.

While investigating the mutation spectrum in adenoma and adenocarcinoma, we found that C: G->T: A transitions were the most significant changes in both adenoma and adenocarcinoma (Figure 1A). This feature of colorectal cancer was previously reported [10]. Our data show a significantly higher enrichment of C: G->T: A in 5′-CpG-3′ dinucleotides in both adenoma (p = 2.78E-09) and carcinoma (p = 3.14E-54) compared to that expected by chance, respectively (Figure 1B). Given the fact that DNA methylation occurs almost exclusively within the context of CpG dinucleotides [23], the enrichment of C:G->T:A in 5′-CpG-3′ dinucleotides may be associated with the extensive methylation of 5′-CpG-3′ dinucleotides in colon cancer [24], [25].

The mutation rate in the adenoma is different from that in the adenocarcinoma of this case (Figure 1C). The adenoma has a much lower mutation rate in both the coding region (0.94 vs. 2.91 mutations/Mb, p = 1.98E-06) and the overall sequencing region (0.91 vs. 1.93 mutations/Mb, p = 1.75E-09) compared with the adenocarcinoma. The coding region in the adenocarcinoma has a significantly higher mutation rate than the overall sequencing region (2.91 vs. 1.93 mutations/Mb, p = 0.0045). However, the mutation rate of the coding region in the adenoma is similar to that of the overall sequencing region (0.94 vs. 0.91 mutations/MB, p = 0.995).

### Validation of Mutations Identified in the Adenoma and the Adenocarcinoma from the same Patient

Nonsynonymous SNVs were validated by Sanger sequencing in combination with Sequenom genotyping. Taken together, 12 SNVs (75.0%) out of 16 nonsynonymous somatic SNVs in the adenoma were confirmed (Table 2); and 42 SNVs (95.4%) out of 44 nonsynonymous somatic SNVs in the adenocarcinoma were confirmed (Table 3). Our result is consistent with previous reports showing that the number of nonsynonymous SNVs in the coding region of CRC is between 7 and 113 [10]–[12]. Among these mutated genes, mutations in APC, FBXW7, KIAA1409, COL4A6, FAM181A, TFR2 and NRXN3 were previously reported in colon adenomas or adenocarcinomas [10]–[12]. In addition, 5 out of 12 mutated genes identified in our cases of adenoma and 15 of 42 mutated genes identified in our cases of adenocarcinomas were reported previously to have genetic mutations in non-colorectal cancers [26]. Genetic alterations in the remaining mutated genes have not been reported in the past in colorectal cancer or any other malignancies.

**Table 2:** Validated somatic mutation in the colorectal adenoma.

Sample	Gene	Genomic Change	Amino acid	Reported[10]–[12], [26]
Adenoma	OR6X1	g.chr11:123129881C->T	p.A186T	
Adenoma	SLC15A3	g.chr11:60461415A->G	p.I533T	
Adenoma	KRTHB4	g.chr12: 51061435C->T	p.A352T	
Adenoma	RBFOX1	g.chr16: 7569894C->T	p.R149W	
Adenoma	LAMA3	g.chr18: 19762624T->C	p.L2778S	*
Adenoma	CDH20	g.chr18: 57354795G->A	p.R454Q	*
Adenoma	BIRC6	g.chr2: 32494045G->T	p.E728X	*
Adenoma	APC	g.chr5: 112203247G->T	p.E1353X	*
Adenoma	NMBR	g.chr6: 142438569C->T	p.R361H	*
Adenoma	GLCCI1	g.chr7: 8092565C->T	p.L506F	
Adenoma	EFR3A	g.chr8: 133057465G->A	p.G390E	
Adenoma	FTHL17	g.chrX: 30999856C->T	p.D46N	

**Table 3:** Validated somatic mutation in the colorectal adenocarcinoma.

Sample	Gene	Genomic Change	Amino acid	Reported[10]–[12], [26]
Adenocarcinoma	PGM1	g.chr1: 63886845 G->A	p.R405H	*
Adenocarcinoma	DTL	g.chr1:210341029G->A	p.G692R	
Adenocarcinoma	PPP1R3C	g.chr10: 93380476 G->A	p.R48X	
Adenocarcinoma	OR51E2	g.chr11: 4660017 G->T	p.F167L	
Adenocarcinoma	RRP8	g.chr11:6579205A->G	p.S223P	
Adenocarcinoma	NARS2	g.chr11:77963118G->A	p.H22Y	
Adenocarcinoma	FAM109A	g.chr12:110285370C->T	p.R95H	
Adenocarcinoma	FAM181A	g.chr14: 93461390 G->T	p.R7I	*
Adenocarcinoma	NRXN3	g.chr14:78245443T->C	p.F78S	*
Adenocarcinoma	KIAA1409	g.chr14:93242879C->T	p.P2418L	*
Adenocarcinoma	TGM7	g.chr15: 41358655 C->T	p.M597I	
Adenocarcinoma	CORO1A	g.chr16: 30105713 C->T	p.R133C	
Adenocarcinoma	KRTHA1	g.chr17: 36805323 G->A	p.R223W	
Adenocarcinoma	GNAL	g.chr18:11743892G->A	p.R191Q	
Adenocarcinoma	PPAP2C	g.chr19: 238556 C->A	p.D134Y	*
Adenocarcinoma	FLJ37549	g.chr19: 42852592 G->A	p.R100W	
Adenocarcinoma	RYR1	g.chr19: 43768631 G->A	p.E4977K	
Adenocarcinoma	GSK3A	g.chr19: 47429297 T->C	p.Q328R	
Adenocarcinoma	ATF2	g.chr2: 175691277 G->A	p.A89V	*
Adenocarcinoma	ZFP36L2	g.chr2:43305936C->A	p.G171C	
Adenocarcinoma	NFATC2	g.chr20: 49525428 C->A	p.L503F	
Adenocarcinoma	ZFP64	g.chr20: 50202574 C->T	p.V468I	
Adenocarcinoma	GRIK1	g.chr21: 29945440 C->T	p.V275I	
Adenocarcinoma	KRTAP19-7	g.chr21:30855383G->A	p.R33C	
Adenocarcinoma	CESK1	g.chr22: 15453028 G->A	p.T138M	
Adenocarcinoma	KLHL22	g.chr22: 19126407 T->C	p.M620V	
Adenocarcinoma	ALPK1	g.chr4: 113573037 C->T	p.P962L	*
Adenocarcinoma	FBXW7	g.chr4: 153471357 G->A	p.R367X	*
Adenocarcinoma	APC	g.chr5: 112179160 C->T	p.R302X	*
Adenocarcinoma	APC	g.chr5: 112203106 G->T	p.E1306X	*
Adenocarcinoma	POU4F3	g.chr5:145700003C->T	p.R274W	
Adenocarcinoma	FLT4	g.chr5:179989704G->A	p.S174L	*
Adenocarcinoma	FAM54A	g.chr6: 136602344 C->A	p.L274F	
Adenocarcinoma	KIF25	g.chr6: 168185641 C->T	p.R264X	
Adenocarcinoma	SDK1	g.chr7: 4085690 G->A	p.R1091Q	
Adenocarcinoma	TFR2	g.chr7:100063331A->G	p.F552L	
Adenocarcinoma	CUX1	g.chr7:101631839G->A	p.G848S	
Adenocarcinoma	NRG1	g.chr8:32733489G->A	p.R123H	
Adenocarcinoma	OR13J1	g.chr9:35860034C->T	p.R122H	*
Adenocarcinoma	COL4A6	g.chrX:107305660C->T	p.V904I	*
Adenocarcinoma	WDR44	g.chrX:117411223G->A	p.E263K	*

### Mutation Analysis in Additional Cases of Adenoma and Adenocarcinoma

In order to know the prevalence of the above-identified mutations in CRC patients, we analyzed all the 54 SNVs in 73 samples of matched adenoma and adenocarcinoma from the same patients and additional 215 samples of adenocarcinomas by sanger sequencing in combination with sequenome genotyping. We found that the same SNVs of APC and FBXW7 repeatedly occurred in these tumors (Table 4). The E1353X mutation of APC was detected in one additional case of adenoma, and was also reported in colorectal adenocarcinomas [27]. The R302X and E1306X mutations of APC were detected in several other adenocarcinomas. The R367X mutation of FBXW7 was detected in several cases of both adenomas and adenomcarcinomas (Table 4). It should be noted that the remaining cases of adenomas and adenocarcinomas may have mutations in other residues of APC and FBXW7. Similarly, although the remaining SNVs were not detected in any of the 73 cases of adenomas and 288 cases of adenocarcinomas, we cannot exclude the possibility that mutations occur in other regions of these 54 genes.

**Table 4:** Recurrent mutations in 288 additional cases of colorectal tumors.

Gene	Mutation	Frequency	SampleID	Tumor
APC	g.chr5: 112203247 G->T	1/73	10-027	Adenoma
FBXW7	g.chr4: 153471357 G->A	2/73	10-005	Adenoma
			10-006	Adenoma
APC	g.chr5: 112179160 C->T	3/288	10-058	Adenoarcinoma
			10-040	Adenoarcinoma
			252T	Adenoarcinoma
APC	g.chr5: 112203106 G->T	2/288	B7C8	Adenoarcinoma
			K3A5	Adenoarcinoma
FBXW7	g.chr4: 153471357 G->A	1/288	187T	Adenoarcinoma

### Pathway Analysis of Mutated Genes in the Adenoma and the Adenocarcinoma

All the validated, mutated genes were annotated through the KEGG pathway analysis [22], the Gene Ontology analysis [28], and literature review (Table S2). We found that several cellular pathways that shared between adenoma and adenocarcinoma (Table 5). First, APC was mutated in both adenoma and adenocarcinoma; thus this study confirmed that the Wnt pathway was activated in the early stage of colorectal tumor development. Second, our study supported the importance of the cell adhesion pathway in the initial process of colorectal tumor development. We found that CDH20 and LAMA3 were mutated in the adenoma while NRXN3 and COL4A6 were mutated in the adenocarcinoma. The products of all these four genes are involved in cell adhesion. Third, the ubiquitination process was also found to be altered in both adenoma and adenocarcinoma. BIRC6, which was found to be mutated in the adenoma, is an ubiquitin-conjugating enzyme (E2) and involved in the apoptosis process. FBXW7, which was found to be mutated in the adenocarcinoma, is a subunit of ubiquitin ligase (E3) and is involved in the cell cycle regulation. Finally, genes involved in olfactory transduction and neuroactive ligand-receptor interaction pathway were also mutated in both the adenoma and the adenocarcinoma.

**Table 5:** Mutated pathways in adenoma and adenocarcinoma.

Pathways involved in carcinogenesis	Mutated genes in Adenoma	Mutated genes in Adenocarcinoma
Cell adhesion pathway	CDH20; LAMA3	NRXN3;COL4A6
Wnt signaling pathway	APC	APC
Ubiquitin mediated proteolysis pathway	BIRC6	FBXW7
Olfactory transduction pathway	OR6X1	OR13J1; OR51E2; GNAL
Neuroactive ligand-receptor interaction	NMBR	GRIK1
Glycerolipid metabolism		PPAP2C
MAPK signaling pathway		ATF2
Aminoacyl-tRNA biosynthesis		NARS2
Cytokine-cytokine receptor interaction		FLT4
Chemokine signaling pathway		GSK3A
Calcium signaling pathway		GNAL
ErbB signaling pathway		NRG1
Glycolysis/Gluconeogenesis		PGM1
Insulin signaling pathway		PPP1R3C

To study how gene mutations affect the expression of genes belonging to the pathways that were found to be altered genetically in both adenoma and adenocarcinoma, we performed the real time PCR analysis of 12 representative mutated genes among those listed in Table 5. As shown in Figure S1, all 12 genes are consistently downregulated in both the adenoma and the adenocarcinoma comparing to the normal colorectal tissue from the same index patient. This has suggested that the mutations identified in these genes may have led to the downregulation of their gene expression in either adenoma or adenocarcinoma of this patient. Interestingly, even though these genes are not mutated in both adenoma and adenocarcinoma, their expression levels are consistently downregulated in both tissues, suggesting that other mechanisms may have led to the downregulation of gene expression.

## Discussion

Our study, for the first time, described the somatic mutation in the whole exome of a colorectal adenoma. The adenoma had a smaller number of somatic mutations but demonstrating a similar pattern of enrichment in nucleotide transitions and involving similar functional pathways as the adenocarcinoma from the same patient. Identification of these gene alterations and their associated pathways in the adenoma has provided new sights into the molecular process of developmental course from colorectal adenoma to colorectal adenocarcinoma.

We identified 12 somatic nonsynonymous SNVs in the adenoma. The APC gene was the only gene reportedly associated with colon cancer. It was found to be mutated in both adenoma and adenocarcinoma. Four other genes were reported as mutated genes in other types of cancer. First, a missense mutation in LAMA3 in adenoma changed from leucine to serine. LAMA3 is involved in cell adhesion, signal transduction and differentiation and its mutations were previously reported in brain cancer [29], ovary cancer [15] and melanoma [30]. Second, a missense SNV in CDH20 changed from arginine to glutamine. CDH20 encodes a type II classical cadherin which is involved in cell to cell adhesion and its mutation was previously reported in breast cancer [10]. Third, a nonsense mutation was found in BIRC6 and would result in a truncated protein. The mutations in BIRC6 were previously reported in several kinds of cancer including melanoma [31], glioma [29], breast cancer [11] and pancreatic cancer [32]. Its gene product, also known as APOLLON, acts as an ubiquitin-conjugating enzyme (E2) and inhibits apoptosis by facilitating the degradation of apoptotic proteins by ubiquitination. Fourth, a missense SNV was found in NMBR. NMBR encodes a Neuromedin B receptor, and its mutations were previously reported in gliomas [29], [33].

There were 42 somatic nonsynonymous SNVs in the adenocarcinoma. Among them, APC and FBXW7 were reported as two mutated gene ‘mountains’ in colorectal carcinomas [11]. In addition, 5 other mutated genes were previously reported in colon cancers including FAM181A, NRXN3, KIAA1409, TFR2, and COL4A6. The mutation of FAM181A was reported in colon cancers [12]. But its function is not clear, so the further funcational study will be needed. NRXN3 functions in the vertebrate nervous system as cell adhesion molecules and receptors and was previously found to be mutated in colon cancer [12], breast cancer [10], glioma [33], squamous cell carcinoma of the head and neck [34], [35] and ovary cancer [15]. The mutation of KIAA1409, which is an unknown protein, was previously reported in colon cancers [11], ovary cancer [15], and melanoma [30]. TFR2 encodes a single-pass type II membrane protein involved in iron uptake and was previously found to be mutated in colon cancer [12] and liver cancer [36]. COL4A6 encodes one of the six subunits of type IV collagen likely involved in cell to cell adhesion and was found to be mutated in ovary cancer [15] and colon cancer [11]. We observed mutations in other 8 genes in the adenocarcinoma. Their mutations were only reported in non-CRC malignancies. Thus, they are newly discovered gene mutations in CRCs.

In addition to the APC/Wnt pathway known to be involved in the development of the colorectal adenoma, this study has identified a few new cellular pathways that are altered in the adenoma at the genetic level. These pathways are also altered at the genetic level in the adenocarcinoma, suggesting that they may functionally drive colorectal tumorigenesis. First, CDH20 and LAMA3, which are both involved in cell adhesion, were found to be mutated in the colorectal adenoma. This is quite interesting as the cell adhesion pathway is thought to be important for cancer cell invasion and metastasis, which are the characteristics of the later stage of cancer development. Our finding thus provides direct evidence supporting a recently proposed hypothesis that the capacity of metastasis may have been acquired by the cancer cells at the early stage of their development [37]. Second, our result also showed that ubiquitin mediated proteolysis pathway is altered at the genetic level in the early stage of tumorigenesis. BIRC6 which encodes E2 ubiquitin-conjugating enzyme is involved in ubiquitin mediated proteolysis. Although we found that FBXW7 which encodes E3 ubiquitin-ligase enzyme is mutated only in colorectal adenocarcinomas [38], previous report has identified somatic mutations in FBXW7 in adenomas [39]. Recently, whole-exome sequencing of premalignant lesions of pancreatic adenocarcinoma also revealed recurrent mutations in components of ubiquitin-dependent pathways [40], [41]. Our study thus suggested that the ubiquitin mediated proteolysis may play a role in the early stage of tumorigenesis in the CRC. Third, we found OR6X1 and NMBR, which are involved in olfactory transduction and neuroactive ligand-receptor interaction, respectively, are mutated in the adenoma. Many proteins involved in neuronal transduction are also involved in cell-cell communication, which again is important for cancer cell invasion and metastasis. The functions of the mutations of OR6X1 and NMBR in the early stage of colorectal tumorigenesis remain to be explored. Multiple genes involved in olfactory transduction and neuroactive ligand-receptor interaction including OR13J1, OR51E2, GNAL, and GRIK1 were also found by this study to be mutated in the adenocarcinoma, suggesting these two classes of genes warrant further investigation. Finally, although neither the remaining mutations found in the adenoma nor the genetic alterations in their involved pathways are observed in the adenocarcinoma, their importance in the development of colorectal adenoma cannot be excluded.

Although the host environmental factors were largely eliminated by analyzing the mutation profiles of the adenoma and the adenocarcinoma from the same patient, it is not surprising that mutations in the adenoma are not present in the adenocarcinoma from the same patient because they may have arisen from two independent tumorigenesis processes. Nevertheless, we found that mutations affecting the same cellular pathways were found in both the adenoma and the adenocarcinoma from the same patient. It is also not surprising that identical mutations identified in the adenoma from one patient were not found in adenomas from additional patients tested in this study. We anticipate that different adenomas harbor mutations in different regions on the same genes that were found to be mutated in one adenoma. If these genes are important for the development of adenoma, we may see them repeatedly altered in multiple adenomas by sequencing their entire exome. However, none of them except APC are anticipated to be frequently mutated in the adenoma. The same cellular pathways such as the cell adhesion and ubiquitin-dependent pathways may be altered in other components at the genetic level more frequently in the adenomas and therefore warrant further investigation.

## Supporting Information

Figure S1 **mRNA expression of genes whose mutations were identified in either adenoma or adenocarcinoma by the exome capture sequencing.** Gene expression levels were measured by the quantitative real-time PCR analysis on RNA purified from the normal mucosa, adenoma and adenocarcinoma tissue from the same patient, respectively. Exome capture sequencing was performed on these same tissues. Each real-time PRC experiment was repeated for at least three times. The GAPDH expression level was used as an internal control. Expression levels relative to those in normal mucosa after being normalized with the GAPDH expression level are shown in histogram.
(TIF)

Table S1 **The process of selecting somatic nonsynonymous mutations in adenoma and adenocarcinoma.**
(XLS)

Table S2 **Functional annotation and Pathway analysis of somatic mutaiton in adenoma and adenocarcinoma.**
(XLS)

Table S3 **Pathway analysis of differential expressed genes in adenoma.**
(XLS)

Table S4 **Pathway analysis of differential expressed genes in adenocarcinoma.**
(XLS)

Table S5 **Primers for validation of somatic SNVs.**
(XLS)
