# Supplementary figures and images for: Exome Capture Sequencing of Adenoma Reveals Genetic Alterations in Multiple Cellular Pathways at the Early Stage of Colorectal Tumorigenesis
Source: PLoS One. 2013 Jan 2;8(1):e53310. doi: 10.1371/journal.pone.0053310 (PMC3534699; doi:10.1371/journal.pone.0053310)

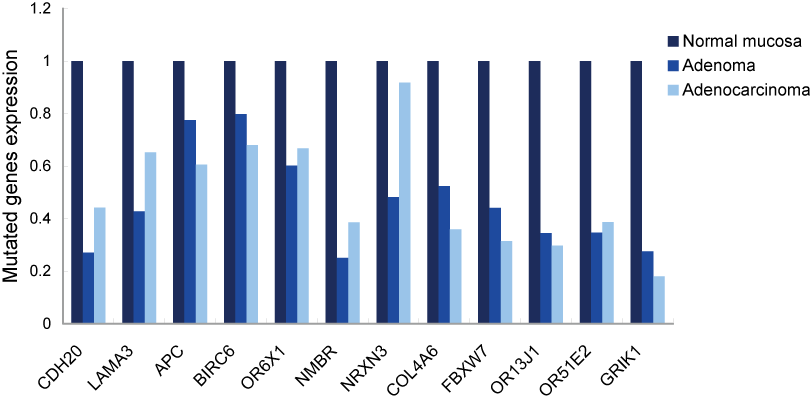

Supplement: Figure S1 — mRNA expression of genes whose mutations were identified in either adenoma or adenocarcinoma by the exome capture sequencing. Gene expression levels were measured by the quantitative real-time PCR analysis on RNA purified from the normal mucosa, adenoma and adenocarcinoma tissue from the same patient, respectively. Exome capture sequencing was performed on these same tissues. Each real-time PRC experiment was repeated for at least three times. The GAPDH expression level was used as an internal control. Expression levels relative to those in normal mucosa after being normalized with the GAPDH expression level are shown in histogram. (TIF) [file pone.0053310.s001.tif]
